# Irgm1 regulates metabolism and function in T cell subsets

**DOI:** 10.1038/s41598-021-04442-x

**Published:** 2022-01-17

**Authors:** Yazan Alwarawrah, Keiko Danzaki, Amanda G. Nichols, Brian E. Fee, Cheryl Bock, Gary Kucera, Laura P. Hale, Gregory A. Taylor, Nancie J. MacIver

**Affiliations:** 1 Department of Pediatrics, Division of Pediatric Endocrinology and Diabetes, University of North Carolina, Chapel Hill, NC USA; 2 Department of Pediatrics, Division of Pediatric Endocrinology and Diabetes, Duke University Medical Center, Durham, NC USA; 3 Department of Medicine, Division of Geriatrics, and Center for the Study of Aging and Human Development, Duke University Medical Center, Durham, NC USA; 4 Geriatric Research, Education, and Clinical Center, Durham VA Health Care System, Durham, NC USA; 5 Duke Cancer Institute, Duke University Medical Center, Durham, NC USA; 6 Department of Pathology, Duke University Medical Center, Durham, NC USA; 7 Department of Molecular Genetics and Microbiology, Duke University Medical Center, Durham, NC USA; 8 Department of Immunology, Duke University Medical Center, Durham, NC USA; 9 Department of Nutrition, University of North Carolina, Chapel Hill, NC USA

**Keywords:** Biochemistry, Immunology

## Abstract

Immunity Related GTPases (IRG) are a family of proteins produced during infection that regulate membrane remodeling events in cells, particularly autophagy and mitophagy. The human *IRGM* gene has been strongly associated with Crohn’s disease and other inflammatory diseases through Genome-Wide Association studies. Absence of *Irgm1* in mice prompts intestinal inflammation, autoimmunity, and impaired immune control of pathogenic bacteria and protozoa. Although prior work has focused on a prominent role for IRGM/Irgm1 in regulating macrophage function, the work described here addresses a potential role of Irgm1 in regulating the function of mature T cells. Irgm1 was found to be highly expressed in T cells in a manner that varied with the particular T cell subset and increased with activation. Mice with a complete lack of Irgm1, or a conditional lack of Irgm1 specifically in T cells, displayed numerous changes in T cell numbers and function in all subsets examined, including CD4^+^ (Th1 and Treg) and CD8^+^ T cells. Related to changes in T cell number, apoptosis was found to be increased in Irgm1-deficient CD4^+^ and CD8^+^ T cells. Altered T cell metabolism appeared to be a key driver of the phenotypes: Glucose metabolism and glycolysis were increased in Irgm1-deficient CD4^+^ and CD8^+^ T cells, and muting these effects with glycolytic inhibitors partially restored T cell function and viability.

## Introduction

Immunity Related GTPases (IRG) constitute a family of dynamin-like proteins that are produced at high levels in cells exposed to interferon (IFN)-γ or lipopolysaccharides (LPS)^1^. IRGs play important roles in inflammatory disease, as allelic variants of human *IRGM* are strongly associated with overall incidence^2,3^, ileal involvement^4^, fistulating behavior^5^, and need for surgery^6^ in Crohn’s disease (CD), and are associated with other diseases including *Mycobacterium tuberculosis* infection^7,8^, sepsis^9^, and non-alcoholic fatty liver disease^10^. One function ascribed to IRGM—and to a mouse orthologue, Irgm1—is positive regulation of autophagy, including autophagic removal of pathogenic bacteria from host cells such as macrophages^11–13^. IRGM/Irgm1 have also been found to regulate autophagic removal of mitochondria (mitophagy)^14–19^. In absence of IRGM/Irgm1, deficient mitochondrial homeostasis leads to engagement of the cGAS/STING and TLR7 pathways leading to type I IFN production^16,19^. Control of bacterial homeostasis and cytokine production in macrophages are thought to be primary mechanisms through which IRGM/Irgm1 control inflammatory diseases such as CD.

CD is characterized by aberrant functioning of epithelium and/or complex immune cell populations in the intestinal lamina propria. While macrophages play important roles in the lamina propria, T cells are clearly pivotal in maintaining inflammatory homeostasis. Effector CD4^+^ T cells (Teff) drive productive inflammatory responses that coordinate defense against pathogens, whereas regulatory T cells (Treg) limit the expansion and overactivity of CD4^+^ Teff^20^. A robust literature indicates that inflammatory bowel disease can result from either an excessive activation of Teff^21^, or from deficits in immunosuppression by Treg cells^22,23^. Although past reports have hinted that IRGM/Irgm1 may influence T cell function, how this may occur is not clear. One study found that Irgm1 regulates homeostasis of CD3^+^ T cells during chronic mycobacterial infection by driving expansion of bone marrow progenitor cells, likely as a consequence of the ability of Irgm1 to regulate autophagy^24–26^. A more recent study found a decrease in Th1 cells in the lamina propria of *Irgm1*^−/−^ mice that were infected with the enteric bacterium *Citrobacter rodentium*, though the underlying mechanism was not addressed, and it was not clear whether that was a primary or secondary consequence of Irgm1-deficiency^27^. Thus, there is a need to understand how IRGM/Irgm1 may regulate the development, homeostasis, and function of mature T cell subsets.

Over the last decade, it has become clear that T cell function is closely linked with T cell metabolism. Upon activation, CD4^+^ Teff cells (including Th1, Th2 and Th17 cells) and CD8^+^ cytotoxic T cells have been found to upregulate glucose metabolism (glycolysis) to promote growth, proliferation, and effector function in a manner dependent upon increased expression of the glucose transporter Glut1^28–32^. In contrast, naïve T cells, regulatory T cells (Treg), and memory T cells predominantly utilize fatty acid oxidation to fuel suppressive function and immune surveillance^33–36^. However, the role of fatty acid oxidation in regulating T cell differentiation and function is nuanced^37^; for example, some lipid metabolites such as selected oxysterols are important for Th17 differentiation^38^. A number of studies have demonstrated that the regulation of nutrient uptake and utilization is critically important for the control of T cell subset differentiation and function^28,29,32^. A connection was recently established between Irgm1 and metabolic regulation, specifically that Irgm1-deficiency leads to enhanced glucose metabolism causing an increase in inflammatory cytokine production^15^. That study, however, was performed in macrophages, and thus, it is unknown whether IRGM/Irgm1 may similarly regulate the metabolism of T lymphocytes or other cells.

To address the lack of knowledge of IRGM/Irgm1 function in T cells, we performed a detailed analysis of Teff and Treg function in the absence of Irgm1. We found that Irgm1 does indeed regulate the function of mature T cells, in a subset-specific manner, and it appears to do so by impacting T cell metabolism. These results may have mechanistic implications for IRGM as a regulator of inflammatory diseases such as CD.

## Methods

### Animals

*Irgm1*^−/−^ mice have been described previously^39,40^. They have been backcrossed to C57BL/6NCr1 mice for 9 generations and were maintained by breeding *Irgm1*^+/−^ mice, with the resulting *Irgm1*^+/+^ and *Irgm1*^−/−^ littermate mice used in these experiments. *Irgm1*^fl/fl^ mice were created by first using BAC recombineering to create an *Irgm1* targeting vector. The third exon in the *Irgm1* gene was flanked with Cre recombinase-specific homologous recombination sites (loxP) inserted inside AvlII and PvuII restriction recombinase sites. This vector was electroporated into embryonic stem cells, and recombinants selected. Targeted ES cells were injected into mouse morulae to create chimeric mice^41^, which were bred to homozygosity, backcrossed to C57BL/6 J mice for 8 generations and then crossed with T cell-deleted *Lck-Cre*^+^ recombinase mice (#012837, Jackson Labs, Bar Harbor ME). All mice were housed under specific pathogen free (SPF) conditions and given ad libitum access to food and water. All mouse studies were approved by the Institutional Animal Care and Use Committees of Duke University Medical Center and the Durham VA Medical Center in accordance with the Public Health Service Policy on the Human Care and Use of Laboratory Animals under the United States of America National Institutes of Health (NIH) Office of Laboratory Animal Welfare (OLAW). All studies were carried out in compliance with ARRIVE guidelines.

### T cell isolation

To isolate CD4^+^ or CD8^+^ T cells, spleens were dispersed into single cell suspension in serum-free RPMI 1640 media (Corning, Corning, NY) by pressing the mashed spleen through a 70 μm strainer using a 3 ml syringe plunger. RBCs were lysed by incubating splenocytes in 1–2 ml of *ACK* (Ammonium-Chloride-Potassium) Lysing Buffer for 2 min. Complete RPMI 1640 media was then added, and the cells were collected by centrifugation at 1400 rpm for 5 min. Cells were then resuspended in PBS containing 2 mM EDTA and 0.5% BSA, and CD4^+^ or CD8^+^ T cells were isolated by magnetic negative selection (Miltenyi Biotec, Auburn, CA; StemCell technologies, Cambridge, MA) following manufacturer protocols. Cells were activated by plate-bound 5 μg/ml anti-CD3 and 5 μg/ml anti-CD28 antibodies (eBioscience, Fisher Scientific, Hampton, NH) for 48 h, then collected for further analysis. For the isolation of natural Treg cells, isolated CD4^+^ T cells were subjected to magnetic positive selection for CD25^+^ cells (Miltenyi Biotec) following the manufacturer protocol.

### Flow cytometry

To identify Treg, Th1 and Th17 cells, the following antibodies were used: APC Rat anti-mouse Foxp3 (Biolegend, San Diego, CA), APC Rat anti-mouse RORγt (eBioscience), PE mouse anti-mouse T-bet (Biolegend), BV605 Armenian Hamster anti-mouse CD3e (BD BioSciences, Franklin Lakes, NJ), Pacific Blue rat anti-mouse CD4 (Biolegend), and Alexa fluor 488 rat anti-mouse CD25 (Biolegend). Between 500,000 and one million splenocytes were incubated in a 96 well v-bottom plate in a volume of 100 μl of FACS buffer (2% FBS in PBS) containing rat anti-mouse CD16/CD32 (BD BioSciences) for 30 min at 4 °C. The cells were then washed, fixed and permeabilized using the Foxp3 Transcription Factor Staining Buffer kit (eBioscience) and stained for Foxp3 and GLUT1 (Abcam, Cambridge, United Kingdom) or RORγt and T-bet following the manufacturer instructions. For intracellular cytokines staining of effector T cells (Th1, Th17 and CD8) the following antibodies were used: BV605 Armenian Hamster anti-mouse CD3e (BD BioSciences), Pacific Blue rat anti-mouse CD4 (Biolegend), PE/Cy7 rat anti-mouse CD8a (Biolegend), APC rat anti-mouse IL-17A (eBioscience), APC anti-mouse granzyme B, and FITC rat anti-mouse IFNγ (Biolegend). One million splenocytes were incubated for 4.5 h in complete media containing Golgi Plug (2 μl/ml) (BD Biosciences), *phorbol 12*-*myristate 13*-*acetate* (PMA) (50 ng/ml) (Sigma-Aldrich, St. Louis, MO), and ionomycin (1 μg/ml) (Sigma-Aldrich). The cells were then washed with FACS buffer, stained for surface markers, permeabilized and fixed with Cytofix/Cytoperm kit (BD Biosciences) and stained for IFNγ, IL-17A and GLUT1 (Abcam) following the manufacturer’s protocol. All samples were analyzed on a BD FACSCanto II flow cytometer, and data were processed using the FlowJo software (BD Biosciences).

### In vitro differentiation of CD4^+^ T cells subsets

CD4^+^ T cells were differentiated toward Th1, Th17 and Treg cell subsets as previously described^42^. Briefly, isolated CD4^+^ T cells were cultured for 3 days in the presence of irradiated splenic feeder cells (3000 rad) with 2.5 μg/mL soluble anti-CD3 (Biolegend). The following cytokines were added to each subset—Th1: 10 ng/ml IL-12 (R&D Systems), 10 μg/ml anti–IL-4 (eBioscience); Th17: 20 ng/mL IL-6 (Biolegend), 2.5 ng/ml human TGFβ (R&D Systems), 10 μg/mL anti-IFN-γ, 10 ng/ml IL-23; and Treg: 6 ng/ml human TGFβ. Three days after stimulation, cells were split 1:2 and plated with human IL-2 20 ng/ml (Biolegend) for an additional 2 days, except for Th17 cells which were split without the addition of IL-2.

### Glucose uptake assay

Glucose uptake was determined by monitoring the accumulation of 2-Deoxy-2-[(7-nitro-2,1,3-benzoxadiazol-4-yl)amino]-D-glucose (2NBDG) in T cells as previously described^43^. In brief, cells were washed with glucose- free RPMI media containing 0.5% heat-inactivated FBS then incubated in the same media with 100 μM 2NBDG (Thermo-Fisher). After 30 min, the cells were washed with FACS buffer, stained for surface markers, and analyzed by flow cytometry.

### Glycolysis assay

Glycolytic rate was measured by quantifying the release of ^3^H_2_O from [5-3H]-glucose as previously described^44^. In brief, differentiated Th1, Th17 and Treg cells were collected and washed with glucose-free Krebs buffer (115 mM NaCl, 2 mM KCl, 25 mM NaHCO_3_, 1 mM MgCl_2_, 0.25% BSA pH 7.4) and incubated in 24 well plate at a density of 2 × 10^6^ cells/ml (500 µl/well) in the same buffer for 30 min at 37 °C with 5% CO_2_. After incubation, 1 µCi of [5-3H]-glucose (PerkinElmer, Boston, MA) mixed with 10 mM of glucose was added to each well and incubated for 1 h. The glycolysis reaction was then quenched with 500 µl of 200 mM HCl. 100 µl of the reaction was added to small PCR tubes and placed in scintillation vials containing distilled water, sealed with parafilm, and incubated for 4 days. After that, the PCR tubes were transferred to fresh scintillation vials (undiffused fraction) and scintillation cocktail was added to both the diffused fraction and the undiffused fraction. Radioactivity was measured by liquid scintillation counting.

### Treg suppression assay

Isolated Treg cells (nTreg) or in vitro differentiated Treg cells (iTreg) were cultured at a ratio of 1:4 with CellTrace violet or carboxyfluorescein diacetate succinimidyl ester (CFDA-SE)-labeled CD8^+^ T cells (Thermo-Fisher) with irradiated splenocytes (1:5 ratio) and 3 μg/mL of anti-CD3 antibody. Treg suppression of CD8^+^ T cell proliferation was determined after 72 h by measuring division percentage based on CellTrace violet dilution via flow cytometry.

### Annexin assay

CD4^+^ and CD8^+^ T cells were isolated from spleens of *Irgm1*^*−/−*^ and WT mice and activated for 48 h by plate bound anti-CD3 and anti-CD28 antibodies (5 µg/ml each). Cells were collected and washed with FACS buffer and stained with PE-annexin V and 7-AAD (Biolegend) following the manufacturer protocol.

### Mitotracker Green and TMRE staining

Activated CD8^+^ T cells were washed with FACS buffer and incubated at 37 °C for 25 min in the same buffer containing 50 nM Mitotracker Green (Thermo-Fisher) and 100 nM TMRE (Sigma-Aldrich). After that, the cells were washed and resuspended in FACS buffer containing 100 nM TMRE and 1 µM Sytox blue (Thermo-Fisher). Cells were then analyzed by flow cytometry, quantifying Mitotracker green and TMRE fluorescent intensity in Sytox blue negative cells.

### Quantitative RT-PCR

RNA was isolated from CD4^+^ and CD8^+^ T cells and T cell subsets using the RNeasy RNA extraction kit (Qiagen, Germantown,MD), after which cDNA was synthesized using the iScript cDNA synthesis kit (BioRad, Hercules, CA). To detect *Irgm1* expression, RT-PCR reactions were carried out in 384 well PCR plates in a total volume of 4 µl containing 10 µM of Irgm1 reverse and forward primers (Reverse 5′-GCTCCTACTGACCTCAGGTAAC-3′, Forward 5′-TGCTCCACTACTCCCCAACAT-3′), 2 µl of SensiFAST Sybr lo ROX RT-PCR mix (Bioline, Boston, MA), and 5 ng of template cDNA (1 µl). The reaction was run on a QuantStudio 5 RT-PCR thermo cycler (Thermo-Fisher).

### Cytokine determination by ELISA

Enzyme-linked immunosorbent assays (ELISA) were performed on supernatants from T cell cultures using Invitrogen IFN-γ Mouse ELISA Kit (Thermo-Fisher), IL-17 Mouse ELISA Kit (Biolegend), and granzyme B Mouse ELISA Kit (Biolegend) per the manufacturer’s instructions.

### Western blot

Resting, activated, and differentiated T cells from WT mice were washed with ice cold PBS and lysed with RIPA buffer (Sigma-Aldrich). After protein measurement using the Bio-Rad DC protein determination kit (Bio-Rad), 50 μg of protein were resolved on a Bio-Rad TGX 8–16% gel (Bio-Rad) and transferred to PVDF membrane for 18 h with a constant voltage of 25 V. Membranes were then blocked with 5% BSA and probed with mouse anti-Irgm1 monoclonal antibody (1B2)^45^ (Millipore-Sigma), rabbit anti-mouse LC3B antibody (2775S, Cell Signaling Technologies), or rabbit anti-mouse β-actin (Cell Signaling Technology, Danvers, MA). Images were acquired using ChemiDoc MP imaging system (Bio-Rad). Band intensities were quantified using image Lab software (Bio-rad). Relative intensity was calculated by dividing Irgm1 or LC3B band adjusted volume intensity by β-Actin band adjusted volume intensity.

### Lactate assay

Media from activated CD4^+^ and CD8^+^ T cell culture was diluted 200 times in lactate assay buffer (100 mM Tris HCl, 20 mM KCl pH 8.5) and 50 μl of the diluted sample was added to 96 well plate. To each sample or lactate standard (0–50 μg/ml), 50 μl of detection reagent was added (2.25 U/ml lactate dehydrogenase (Sigma-Aldrich), 2.7 U/ml Diaphorase (Innovative research, Novi, MI), 2 mM NAD (Sigma-Aldrich), and 28 μM Resazurin (Sigma-Aldrich)) in lactate assay buffer. After incubation for 30 min at 37 °C, fluorescence was measured at EX: 531/EM: 590 nm) using a Vector 3 plate reader (PerkinElmer).

### Data analysis

Grouped plot graphing and t-test statistical analyses were performed using GraphPad Prism 9 (GraphPad Software, Inc., La Jolla, CA). Data were checked for normality using the Shapiro–Wilk test. If the data were normally distributed the unpaired t-tests with Welch’s correction was used; if more than two groups are present, equal standard deviation was not assumed and the Welch ANOVA test with multiples T-test and Welch’s correction was used. For samples that failed the normality test, the nonparametric Mann Whitney test was used; if more than two groups are present, the Kruskal–Wallis test was used with Dunn’s multiple comparisons test to compare group pairs. *p* < 0.05 was determined as significant.

## Results

### Irgm1 is expressed in T cell populations and its deficiency is associated with changes in T cell proportions

**Figure 1:**
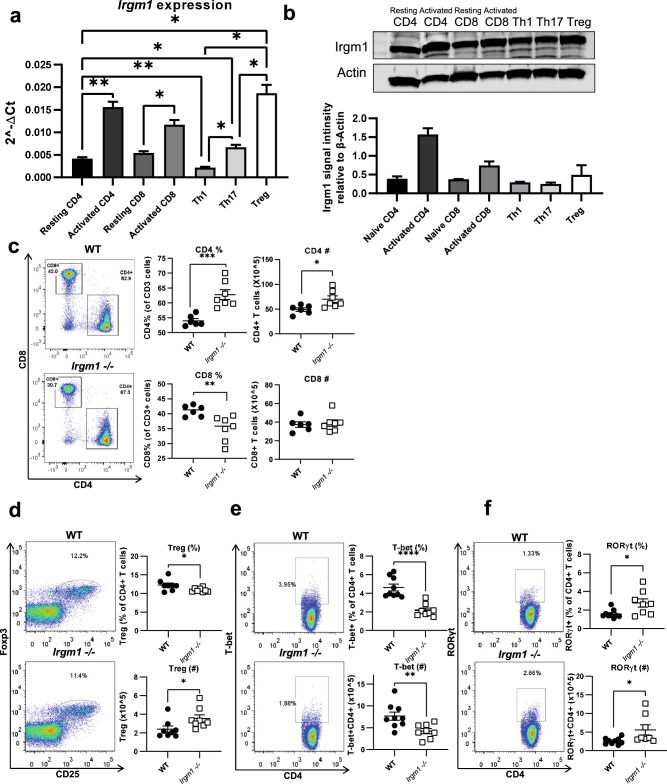
Irgm1 is expressed in T cell populations and its deficiency is associated with changes in T cell proportions. CD4^+^ and CD8^+^ T cells were isolated from spleens of C57BL/6 mice and analyzed at rest or activated with plate bound antibodies to CD3 and CD28. CD4^+^ T cells were further differentiated into Th1, Th17, and Treg cell populations, as detailed in the Methods. (**a**) T cell RNA was generated and *Irgm1* gene expression was analyzed by qRT-PCR. (Welch's ANOVA test *p* = 0.0002, multiple unpaired t with Welch's correction used to compare pairs) (**b**) T cell lysates were generated and immunoblotted for Irgm1 protein expression relative to actin (Kruskal–Wallis test *p* = 0.065, Dunn's multiple comparisons used to compare pairs). (**c-f**) Splenocytes were isolated from WT and *Irgm1*^*−/−*^ mice, stained and analyzed by flow cytometry for CD4 and CD8 (*c*), CD4^+^Foxp3^+^ (Treg) (*d*), CD4^+^T-bet^+^ (Th1) (*e*)* and* CD4^+^RORγt^+^ (Th17) (*f*). Each dot represents a single mouse. (a-c) data are representative of two independent experiments (d-f) data are pooled from 2–3 independent experiments (*n* = *8–9*); error bars represent ± SEM. Mann Whitney test or T-test with Welch’s correction was used to compare groups depending on the normality of the distribution as judged by the Shapiro–Wilk test.. *p < 0.05, **p < 0.01, ***p < 0.001, ****p < 0.0001.

To determine a role for *Irgm1* in T cell function, we first asked if Irgm1 was expressed in T cells and/or T cell subsets in wildtype (WT) C57BL/6 mice. We found that *Irgm1* mRNA was expressed in both CD4^+^ and CD8^+^ T cells, and expression increased following activation (Fig. 1a). We also found that regulatory T cells (Treg cells) expressed more *Irgm1* mRNA compared to T helper 1 (Th1) and T helper 17 (Th17) cells (Fig. 1a). We performed western blotting to confirm the expression of Irgm1 protein in these T cell populations (Fig. 1b).

We then compared the numbers and proportions of T cells and T cell subsets in spleens from WT versus *Irgm1*^−/−^ mice to determine the effect of Irgm1 expression on T cell numbers and differentiation. Using flow cytometry, we found increases in the proportion and number of CD4^+^ T cells from *Irgm1*^*−/−*^ mice and a decrease in the proportion of CD8^+^ T cells from *Irgm1*^*−/−*^ mice (Fig. 1c). Moreover, examination of thymocyte populations in *Irgm1*^−/−^ versus WT control mice showed small increases in single positive CD4^+^ and CD8^+^ thymocytes, and mild decreases in the proportion of CD4^−^CD8^−^ double negative and CD4^+^CD8^+^ double positive thymocytes from *Irgm1*^−/−^ mice compared to WT mice (Supplementary Fig. 1a,b). We next examined CD4^+^ T cell subsets in the spleen and found a decrease in Treg cell proportion in *Irgm1*^−/−^ mice compared to WT mice, as judged by the proportion of cells expressing both Foxp3 and CD25 (Fig. 1d). We also found a decrease in both proportion and number of Th1 cells, defined as CD4^+^ T cells expressing the Th1-specific transcription factor T-bet (Fig. 1e), as well as an increase in the proportion of Th17 cells, defined as CD4^+^ T cells expressing the Th17-specific transcription factor RORγt, in *Irgm1*^−/−^ mice compared to WT controls (Fig. 1f). These findings suggest that Irgm1 expression can influence T cell number, differentiation, and/or survival.

### Irgm1 deficiency is associated with effector and regulatory T cell dysfunction

**Figure 2:**
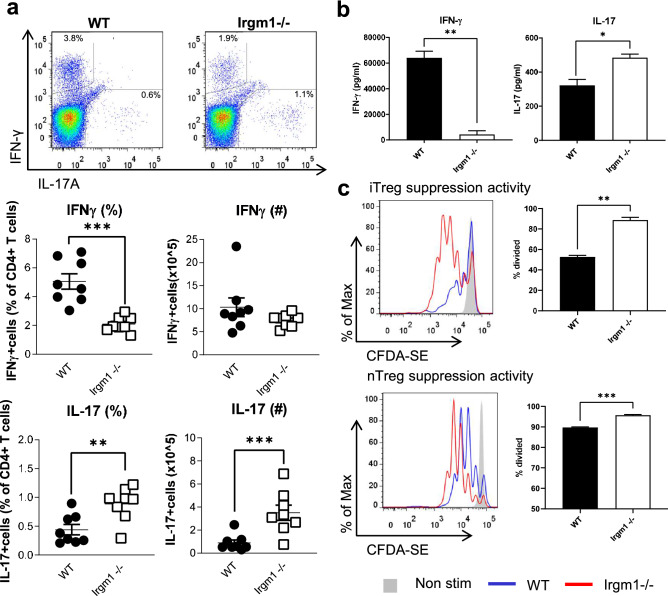
Irgm1 deficiency is associated with effector and regulatory CD4^+^ T cell dysfunction. (**a**) Splenocytes were isolated from WT and *Irgm1*^*−/−*^ mice, stained and analyzed by flow cytometry for CD4^+^IFNγ^+^ and CD4^+^IL-17^+^ Cells. (**b**) CD4^+^ T cells were isolated and activated for 48 h with plate bound anti-CD3 and anti-CD28 antibodies, after which the media was collected and IFNγ and IL-17A were measured by ELISA. (**c**) Isolated induced Treg cells or natural Treg cells from WT and *Irgm1*^*−/−*^ mice were co-cultured for three days with WT CD8^+^ T cells stained with CFDA cell tracer. Treg suppressive capacity was analyzed by the ability to reduce proliferation of CD8^+^ T cells, as measured by the dilution of the fluorescent dye flow cytometrically (The CD8:Treg ratio for the data shown is 1:1 for iTregs and 1:5 for nTregs). Each dot represents a single mouse. (a) data pooled from two independent experiments (*n* = *8*), (b,c) data are representative of two independent experiments (*n* = *3–6*); error bars represent ± SEM. Mann Whitney test or T-test with Welch’s correction was used to compare groups depending on the normality of the distribution as judged by the Shapiro–Wilk test. *p < 0.05, **p < 0.01, ***p < 0.001. ****p < 0.0001.

To gain insight into the importance of Irgm1 for T cell function, we first examined CD4^+^ Teff cytokine production. Using intracellular cytokine staining and flow cytometry, we found that CD4^+^ T cells from *Irgm1*^*−/−*^ mice had lower number and proportion of cells expressing IFNγ and higher number and proportion of cells expressing IL-17A compared to those cells from WT mice (Fig. 2a). We also examined IFNγ and IL-17A production by CD4^+^ T cells by measuring cytokine concentration in supernatants of cultured CD4^+^ T cells activated by plate-bound anti-CD3 and anti-CD28 antibodies using ELISA. Consistent with the intracellular flow cytometry results seen in Fig. 2a, we found decreased IFNγ and increased IL-17A production from activated CD4^+^ T cells from *Irgm1*^−/−^ mice compared to WT controls (Fig. 2b). To assess Treg cell function, we examined the ability of Treg cells to suppress the proliferation of WT CD8^+^ T cells upon activation in vitro using both natural Treg cells (nTreg) isolated from WT and *Irgm1*^−/−^ mice and induced Treg cells (iTreg) that we generated in vitro using activation with plate bound antibodies in the presence of TGF-β. We found that both iTreg cells and nTreg cells from *Irgm1*^−/−^ mice were less effective in blocking the proliferation of activated CD8^+^ T cells compared to Treg cells from WT control mice (Fig. 2c), and therefore had decreased suppressive capacity.

**Figure 3:**
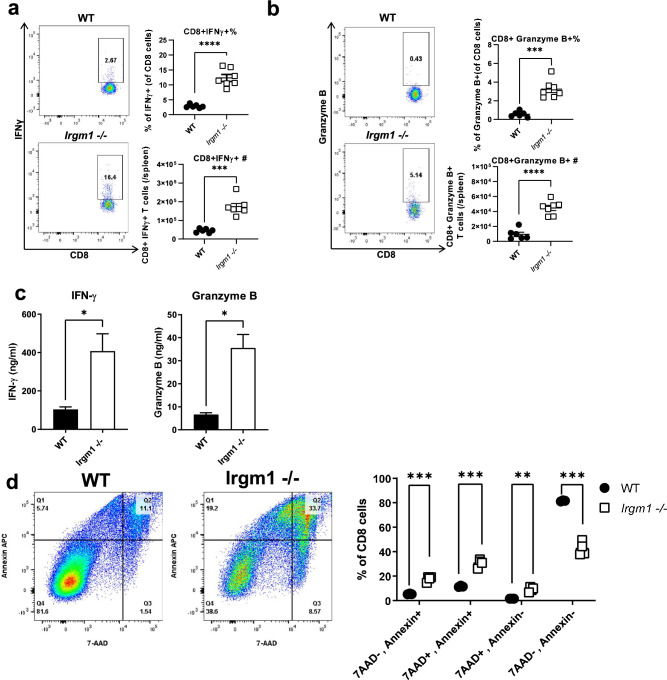
Irgm1 deficiency is associated with CD8^+^ T cell cytokine overproduction and increased apoptosis. (**a-b**) Splenocytes were isolated from WT and *Irgm1*^*−/−*^ mice, stained and analyzed by flow cytometry for CD8^+^IFNγ^+^ (*a*) and CD8^+^Granzyme B^+^ (*b*). (**c**) CD8^+^ T cells were isolated and activated for 48 h with plate bound anti-CD3 and anti-CD28 antibodies after which the media was collected and IFNγ and Granzyme B were measured by ELISA. (**d**) Activated CD8^+^ T cells were assayed for the presence of apoptotic cells, measuring 7-AAD and annexin V positive cells by flow cytometry. (a,b) Data pooled from two independent experiments (*n* = *6–7*), (c, d) data representative of two independent experiments (*n* = *3–4*); error bars represent ± SEM. Mann Whitney test or T-test with Welch’s correction was used to compare groups depending on the normality of the distribution as judged by the Shapiro–Wilk test. *p < 0.05, **p < 0.01, ***p < 0.001, ****p < 0.0001.

We next examined CD8^+^ T cell effector function by measuring the number and proportion of CD8^+^ T cells producing effector cytokines associated with CD8^+^ T cell function, using intracellular cytokine staining and flow cytometry. We found a striking increase in the proportion and number of CD8^+^ T cells expressing both IFNγ and granzyme B from *Irgm1*^−/−^ mice compared to CD8^+^ T cells from WT mice (Fig. 3a,b). Similarly, we found a striking increase in the concentration of both IFNγ and granzyme B in supernatants of activated CD8 + T cells from *Irgm1*^*−/−*^ mice compared to CD8^+^ T cells from WT control mice, as measured by ELISA (Fig. 3c). However, despite the finding that CD8^+^ T cells from *Irgm1*^*−/−*^ mice seem highly functional with increased cytokine production, they had a striking increase in apoptosis following activation in culture compared to CD8^+^ T cells from WT control mice (Fig. 3d). In comparison, CD4^+^ T cells from *Irgm1*^−/−^ mice also had an increase in apoptosis following activation compared to activated CD4^+^ T cells from WT mice, but this was much less striking than the increase in apoptosis seen in activated *Irgm1*^*−/−*^ CD8^+^ T cells (Supplementary Fig. 2). Altogether, these findings indicate that Irgm1 deficiency alters CD4^+^ and CD8^+^ T cell core functions in a subset specific manner while also increasing cell death.

### Changes in T cell population and function are largely due to intrinsic loss of Irgm1 expression

**Figure 4:**
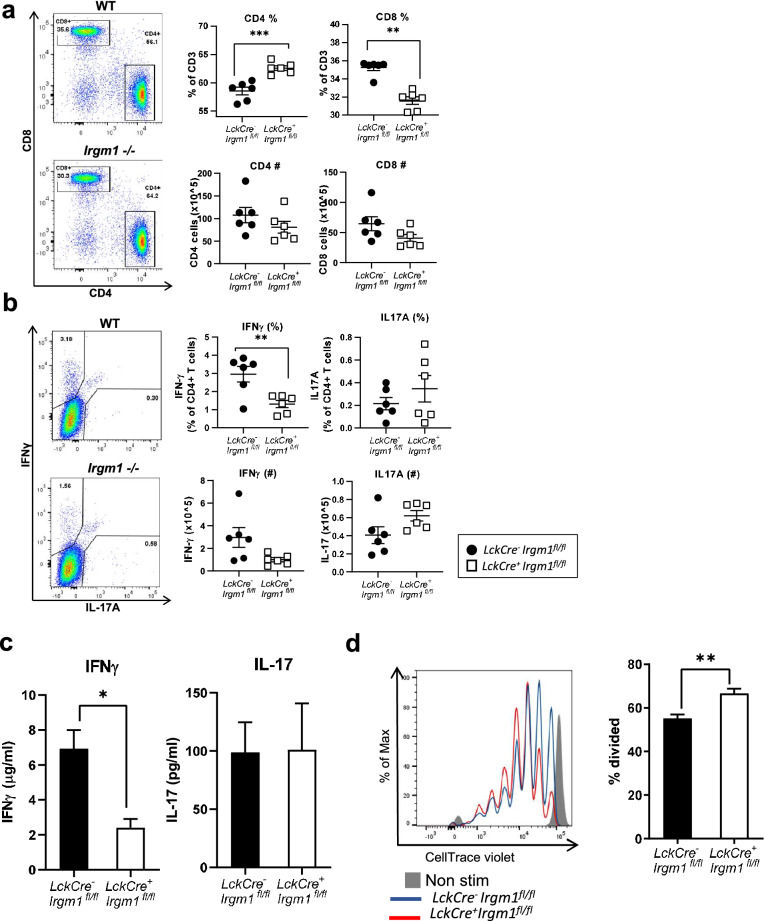
Changes in Th1 and Treg cell populations and function are largely due to intrinsic loss of Irgm1 expression. (**a-b**) Splenocytes were isolated from *LckCre*^+^*Irgm1*^*fl/fl*^ cKO mice and *LckCre*^*−*^*Irgm1*^*fl/fl*^ littermate controls, stained and analyzed by flow cytometry for CD4 and CD8 cells (*a*)*,* CD4^+^IFNγ^+^ and CD4^+^IL-17^+^ (*b*)*.* (**c**) CD4^+^ T cells were isolated and activated for 48 h with plate bound anti-CD3 and anti-CD28 antibodies, after which the media was collected and IFNγ and IL-17A were measured by ELISA. (**d**) In vitro differentiated Treg cells from *LckCre*^+^*Irgm1*^*fl/fl*^ cKO mice and *LckCre*^*−*^*Irgm1*^*fl/fl*^ littermate controls were co-cultured for three days with WT CD8^+^ T cells stained with CellTrace violet. Treg suppressive capacity was analyzed by the ability to reduce proliferation of CD8^+^ T cells, as measured by dilution of fluorescent dye flow cytometrically (The CD8:Treg ratio for the data shown is 2:1). (a, b) data pooled from two independent experiments (*n* = *6*), (c, d) data representative of two independent experiments (*n* = *3–6*); error bars represent ± SEM. Mann Whitney test or T-test with Welch’s correction was used to compare groups depending on the normality of the distribution as judged by the Shapiro–Wilk test.*p < 0.05, **p < 0.01, ***p < 0.001.

A role for Irgm1 in the function of innate immune cells, such as macrophages, has been well described^1^. It is possible that Irgm1-deficiency in myeloid cells may indirectly affect the differentiation and function of T cells. To investigate if the effect on T cells in *Irgm1*^−/−^ mice is due to intrinsic or extrinsic loss of Irgm1 expression, we generated T cell-specific Irgm1 conditional knockout (cKO) mice by crossing *Irgm1*-flox mice expressing Cre under the Lck promoter (*Lck-Cre*^+^) mice, which leads to deletion of *Irgm1* in both CD4^+^ and CD8^+^ T cells. These mice display a greater than 90% decrease in Irgm1 protein expression in splenic T cells (Supplementary Fig. 3). We isolated splenocytes from *Lck-Cre*^+^*Irgm1*^*fl/fl*^ cKO mice and *Lck-Cre*^*−*^*Irgm1*^*fl/fl*^ littermate control mice and determined the numbers and proportions of T cells using extracellular and intracellular flow cytometry. Similar to the global *Irgm1*^*−/−*^ mouse, we found an increase in proportion of CD4^+^ T cells and a decrease in the proportion of CD8^+^ T cells in *Lck-Cre*^+^*Irgm1*^*fl/fl*^ mice compared to littermate controls (Fig. 4a). We also examined the proportions of Th1 and Th17 effector T cells by intracellular cytokine staining for IFNγ and IL-17A using flow cytometry, as a measurement of CD4^+^ T cell effector function. Similar to the global *Irgm1*^*−/−*^ mouse, we found a decrease in IFNγ production in CD4^+^ T cells from *Lck-Cre*^+^*Irgm1*^*fl/fl*^ mice compared to littermate controls, but no significant change in the number or proportion of CD4^+^ T cells producing IL-17A (Fig. 4b). Consistent with the flow cytometry results, we found a decrease in production of IFNγ in the supernatant of activated CD4^+^ T cells from the *Lck-Cre*^+^*Irgm1*^*fl/fl*^ mice compared to the littermate controls, as measured by ELISA, with no significant change in IL-17A concentration (Fig. 4c). To assess Treg cell function, we examined the ability of induced Treg cells from *Lck-Cre*^+^*Irgm1*^*fl/fl*^ mice to suppress the proliferation of WT CD8^+^ T cells in culture. We found that Treg cells from *Lck-Cre*^+^*Irgm1*^*fl/fl*^ mice were less effective in blocking the proliferation of activated CD8^+^ T cells when compared to Treg cells from littermate controls, indicating decreased suppressive capacity (Fig. 4d). Overall, these results show that the majority of the defects in T cell differentiation and function observed with global *Irgm1*-deficiency are T cell-intrinsic (with the exception of altered Th17 cell numbers in global *Irgm1*-deficient mice that appear to be cell extrinsic).

### Irgm1 deficiency in T cells is associated with increased T cell glucose metabolism

Activated T cells have an increased metabolic demand to fuel growth, proliferation, and the production of protein products required for effective immune responses. To meet this increased metabolic demand, activated Teff cells demonstrate a striking increase in glucose uptake and glycolysis, although glutamine metabolism also plays a role^28,46,47^. Moreover, the metabolic profiles of distinct CD4^+^ T cell subsets are nuanced, and select lipid metabolites have been shown to be important in Th17 differentiation^38^. In contrast, resting, memory, and regulatory T cells utilize a largely oxidative metabolism to fuel immune surveillance and suppressive function^33,34,48^, and fatty acid oxidation is important for memory T cell function^49^. Changes in T cell function and metabolism are closely linked: Many studies have now demonstrated that alterations in T cell metabolism can influence T cell number, differentiation, and function^33,48,50^.

**Figure 5:**
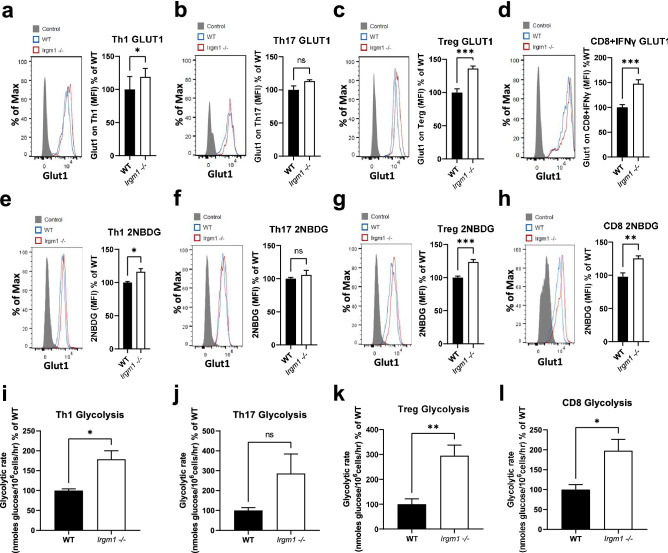
Irgm1 deficiency is associated with increased T cell glucose metabolism. (**a-d**) Splenocytes were isolated from WT and *Irgm1*^*−/−*^ mice, stained and analyzed by flow cytometry for Glut1 expression on CD4^+^IFNγ^+^ (Th1 cells) (*a*), CD4^+^IL-17^+^ (Th17 cells) (*b*)*,* CD4^+^ CD25^+^Foxp3^+^ (Treg cells) (*c*) and activated CD8^+^IFNγ^+^ cells (*d*)*.* (**e–h**) Isolated CD4^+^ and CD8^+^ T cells from WT and *Irgm1*^*−/−*^ mice were differentiated or activated in vitro and uptake of glucose analog 2NBDG was measured flow cytometrically in Th1 (*e*), Th17 (*f*), Treg (*g*) and CD8^+^ T cells (*h*). (**i-l**) Cells were assayed for glycolytic activity by monitoring the formation of tritiated water from glucose in Th1 (*i*), Th17 (*j*), Treg (*k*) and CD8^+^ T cells (*l*). Data pooled from two independent experiments (*n* = *6–8*); error bars represent ± SEM. T-test with T-test with Welch’s correction was used to compare groups after confirming normality using the Shapiro–Wilk test. *p < 0.05, **p < 0.01, ***p < 0.001, ns, not significant.

To gain insight into the role of Irgm1 in regulating T cell metabolism, and thereby influencing function, we first measured protein expression of Glut1, the primary glucose transporter in T cells, using intracellular flow cytometry. We found a significant increase in Glut1 expression in Th1 cells, Treg cells, and activated CD8^+^ cells, but not Th17 cells, from *Irgm1*^*−/−*^ mice compared to WT mice (Fig. 5a–d). Consistent with these results, we also found an increase in glucose uptake, as measured by the uptake of a fluorescent glucose analog in Th1 cells, Treg cells, and activated CD8^+^ T cells, but not Th17 cells, from *Irgm1*^*−/−*^ mice compared to WT mice (Fig. 5e–h). This increase in glucose uptake in *Irgm1* deficiency was accompanied by an increase in glycolytic rate in Th1 cells, Treg cells, and activated CD8^+^ T cells, as measured by the rate of production of tritiated water from tritiated glucose via enolase, the penultimate enzyme in the glycolytic pathway (Fig. 5i–l). Altogether, these results demonstrate a role for Irgm1 in regulating T cell glucose metabolism and provide a possible mechanistic explanation for altered T cell dysfunction in *Irgm1* deficiency. This phenotype has parallels with that of Irgm1-deficient macrophages, which also exhibit a marked glycolytic phenotype^15^ that is thought to be related to alterations in autophagy and mitophagy. In the current study, we found that CD8^+^ T cells from *Irgm1*^*−/− mice*^ demonstrated higher LC3B levels and decreased mitochondrial membrane potential (Supplementary Fig. 4), consistent with prior studies of *Irgm1*^*−/−*^ macrophages^16,51,52^ and suggesting a common core mechanism to explain metabolic alterations in *Irgm1*^*−/−*^ T cells and macrophages.

### Inhibition of glycolysis in *Irgm1*-deficient T cells restores T cell viability and function

**Figure 6:**
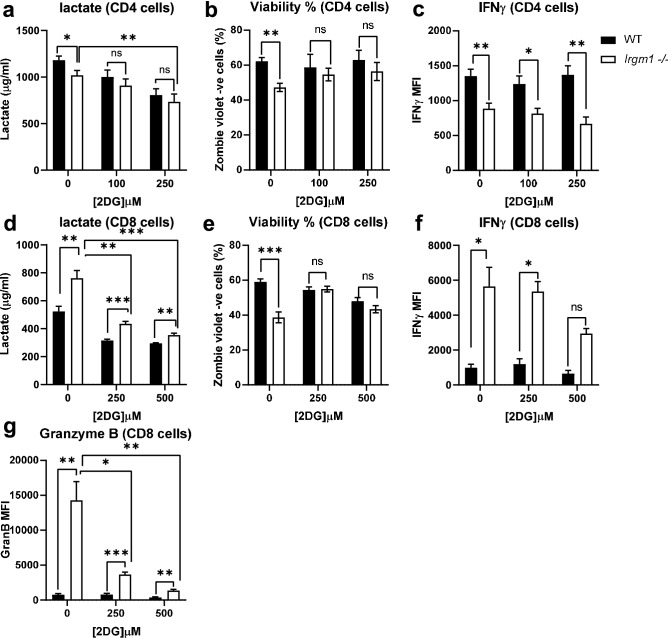
Inhibition of glycolysis in Irgm1 deficient T cells restores T cell viability and function. Isolated CD4^+^ and CD8^+^ T cells from WT and *Irgm1*^*−/−*^ mice were activated in the presence of absence of 2-deoxyglucose (2DG), after which media was collected and assayed for lactate (**a, d**) and cells were stained with Zombie violet dye to assess viability (**b, e**), then fixed, permeabilized and stained for IFNγ (**c, f**) or Granzyme B (**g**). Data pooled from two independent experiments (*n* = *4–6*); error bars represent ± SEM. Dunn's multiple comparisons test or multiple T-test with Welch’s correction was used to compare group pairs depending on the normality of the distribution as judged by the Shapiro–Wilk test *p < 0.05, **p < 0.01, ***p < 0.001, ns, not significant.

To assess the correlation between Irgm1-associated increases in glycolysis and alterations in T cell function, we activated CD4^+^ and CD8^+^ cells in the presence or absence of the glycolytic inhibitor 2-deoxyglucose (2DG) and measured cell viability and cytokine production. As expected, 2DG reduced lactate production in CD4^+^ (Fig. 6a) and CD8^+^ (Fig. 6d) T cell culture media following activation. This was associated with restoration of viability in *Irgm1*^*−/−*^ CD4^+^ and CD8^+^ T cells (Fig. 6b,e), although it was more pronounced in CD8^+^ T cells. While 2DG did not restore IFNγ production from CD4^+^ T cells isolated from *Irgm1*^*−/−*^ mice (Fig. 6c), it partially normalized IFNγ production from CD8^+^ T cells isolated from *Irgm1*^*−/−*^ mice (Fig. 6f). Similar to the effects of 2DG on IFNγ production, we found that activation of CD8^+^ T cells from *Irgm1*^*−/−*^ mice in the presence of 2DG resulted in a striking and significant decrease in granzyme B production (Fig. 6g). Similar results were observed when activating T cells in the presence of the lactate dehydrogenase inhibitor FX11 (Supplementary Fig. 5). These results indicate that the glycolytic phenotype associated with *Irgm1*^*−/−*^ T cells is responsible for changes in CD4^+^ and CD8^+^ T cell viability and in CD8^+^ T cell function.

## Discussion

While prior work has firmly established IRGM/Irgm1 as a positive regulator of macrophage function and homeostasis, the results described here indicate that Irgm1 also profoundly affects homeostasis and function of mature T lymphocytes, for the most part in a cell-autonomous manner. Irgm1 mRNA was found to be highly expressed in T cells, with levels increased about 2–4 fold with activation and being highest in Treg cells compared to other skewed CD4^+^ T cell subtypes. The impact of *Irgm1*-deficiency in T cells varied considerably depending on the specific T cell compartment, with the general trend being toward decreased CD4^+^ T cell function and increased CD8^+^ T cell function. Th1 cells in *Irgm1*^−/−^ mice were present in decreased proportions and numbers, and showed decreased function with diminished IFNγ production. Treg cells showed decreased proportions and suppressive function apparent in both Treg that were expanded in vitro and in, importantly, native Treg isolated from the mice and tested ex vivo. The Th17 compartment was relatively less impacted with undiminished IL-17 production and no change in Th17 numbers that could be attributed to a cell intrinsic effect of Irgm1. In contrast, CD8^+^ T cells from *Irgm1*^−/−^ mice displayed decreased proportions while having striking increases in cytokine production and apoptosis. These phenotypes are conceivably related as cytotoxic effector molecules such Granzyme B and perforin may well act autocrinely to drive CD8 T cell death. The reason for different roles for Irgm1 among T cells subsets was not determined, but may be related to the fact that expression of Irgm1 is greatly upregulated by, and serves as a downstream effector of, IFNγ, a cytokine that plays differing roles among those T cell subsets.

The changes in T cell function and survival with *Irgm1* deficiency were predicated on changes in T cell metabolism that are also driven by absence of Irgm1, namely increased glucose metabolism and increased glycolysis. These findings align with recent work from our lab and others showing increased glycolysis and decreased oxidative metabolism in *Irgm1*^−/−^ macrophages^15,16,51^. For both *Irgm1*-deficient macrophages^15^ and T cells (this paper), muting the increase in glycolysis with the glycolytic inhibitor, 2-deoxyglucose (2DG), suppressed functional changes in cytokine production that are a key part of the *Irgm1*-deficient phenotype, underscoring the pivotal role of those metabolic changes. In the current work, we also found that 2DG reversed the decreased T cell viability that was found to accompany *Irgm1*-deficiency in T cells. Thus, our findings add to and reinforce a growing hypothetical structure suggesting that loss of Irgm1 triggers at least two related pathways: (1) altered mitochondrial metabolism drives cytokine production and apoptotic cell death^15,27^ as demonstrated here, and (2) decreases in clearance of damaged mitochondria prompt mitochondrial DNA engagement of the TLR7 and/ or cGAS/STING pathways driving further cytokine production as shown recently elsewhere^16,19^.

Our results lay a groundwork for future studies into understanding the influence of IRGM/Irgm1 regulation of T cell function and survival in Crohn’s Disease (CD). As alluded to earlier, CD is driven by a breakdown in bacterial homeostasis and/or aberrant inflammatory responses to enteric bacteria, frequently resulting from T cell dysfunction. Our data demonstrate that Th1 CD4 T cells lacking Irgm1 have decreased cell survival as well as deficient function of existing cells, which in combination may well lead to failure to suppress populations of inflammatory enteric bacteria. Along those lines, our recent work has indicated that *Irgm1*-deficient mice are unable to control replication and spread of the enteric bacterium and IBD model, *Citrobacter rodentium*^27^. Our current results also demonstrate that Treg cells lacking Irgm1 have impaired regulatory function, which likely also contributes to inefficient suppression of intestinal inflammatory mechanisms. Future studies should explore these avenues.

## Supplementary Information

Supplementary Information.
