# Supplementary material for: Irgm1 regulates metabolism and function in T cell subsets
Source: Sci Rep. 2022 Jan 17;12:850. doi: 10.1038/s41598-021-04442-x (PMC8763923; doi:10.1038/s41598-021-04442-x)
Supplement: Supplementary file 1 — Supplementary Information. [file 41598_2021_4442_MOESM1_ESM.pdf]

Irgm1 regulates metabolism and function in T cell subsets

Supplemental Material

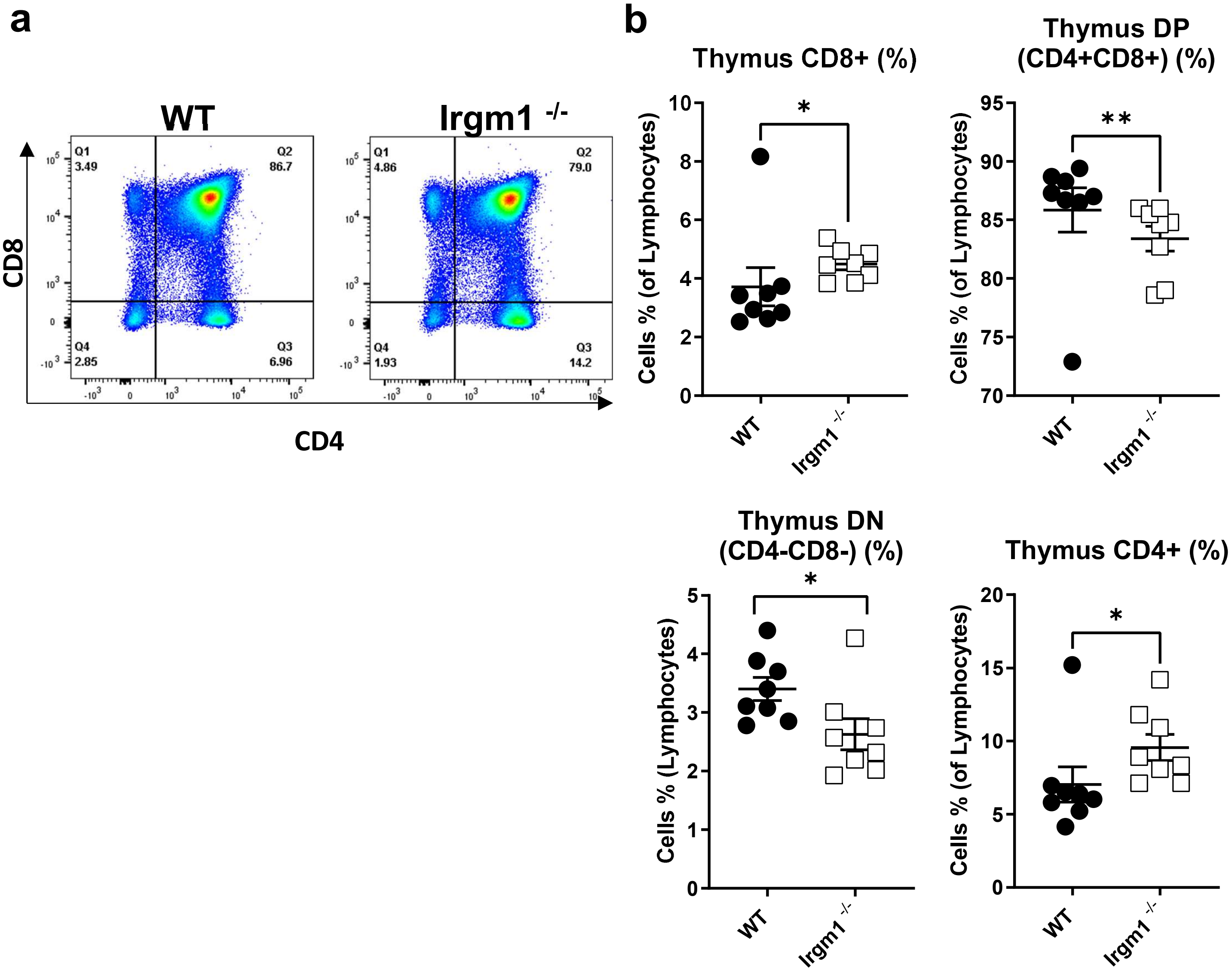

Supplementary Figure 1. Irgm1 deficiency is associated with changes in Thymocytes populations. (a-b) Thymocytes were isolated from WT and *Irgm1*$^{-/-}$ mice and stained and analyzed by flow cytometry for CD4 and CD8. Data is pooled from two independent experiments (n = 8). Mann Whitney test or T-test with Welch's correction was used to compare groups depending on the normality of the distribution as judged by the Shapiro-Wilk test. *p<0.05 **p<0.01.

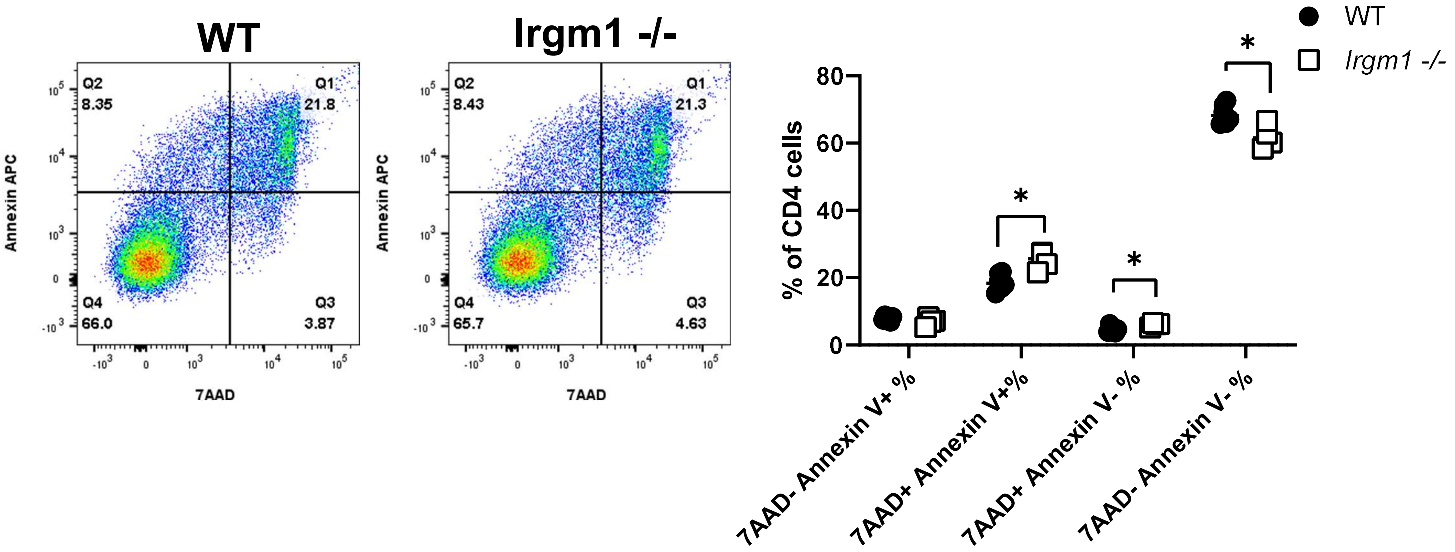

Supplementary Figure 2. Irgm1 deficiency is associated with increased CD4$^+$ T cell apoptosis. CD4$^+$ T cells were isolated from WT and *Irgm1$^{-/-}$* mice and activated for 48 hours with plate bound anti-CD3 and anti-CD28 antibodies. The activated CD4$^+$ T cells were assayed for the presence of apoptotic cells, measuring 7AAD and Annexin V by flow cytometry. Data is representative of two independent experiments (n= 4-6). Normality was tested using the Shapiro-Wilk test and T-test with Welch's correction was used to compare groups. *p<0.05.

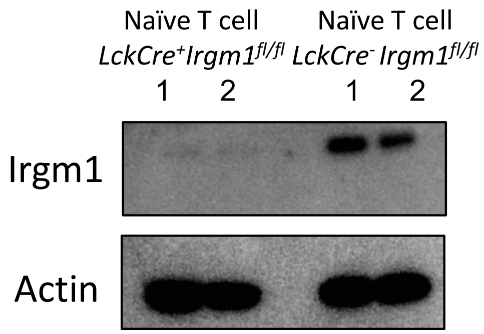

Supplementary Figure 3. Irgm1 expression is more than 90% reduced in T cells from ***LckCre$^+$Irgm1$^{fl/fl}$*** **mice.** T cells were isolated from the spleens of *LckCre$^+$Irgm1$^{fl/fl}$* and *LckCre$^-$Irgm1$^{fl/fl}$* . Cells were lysed and probed for the expression of Irgm1.

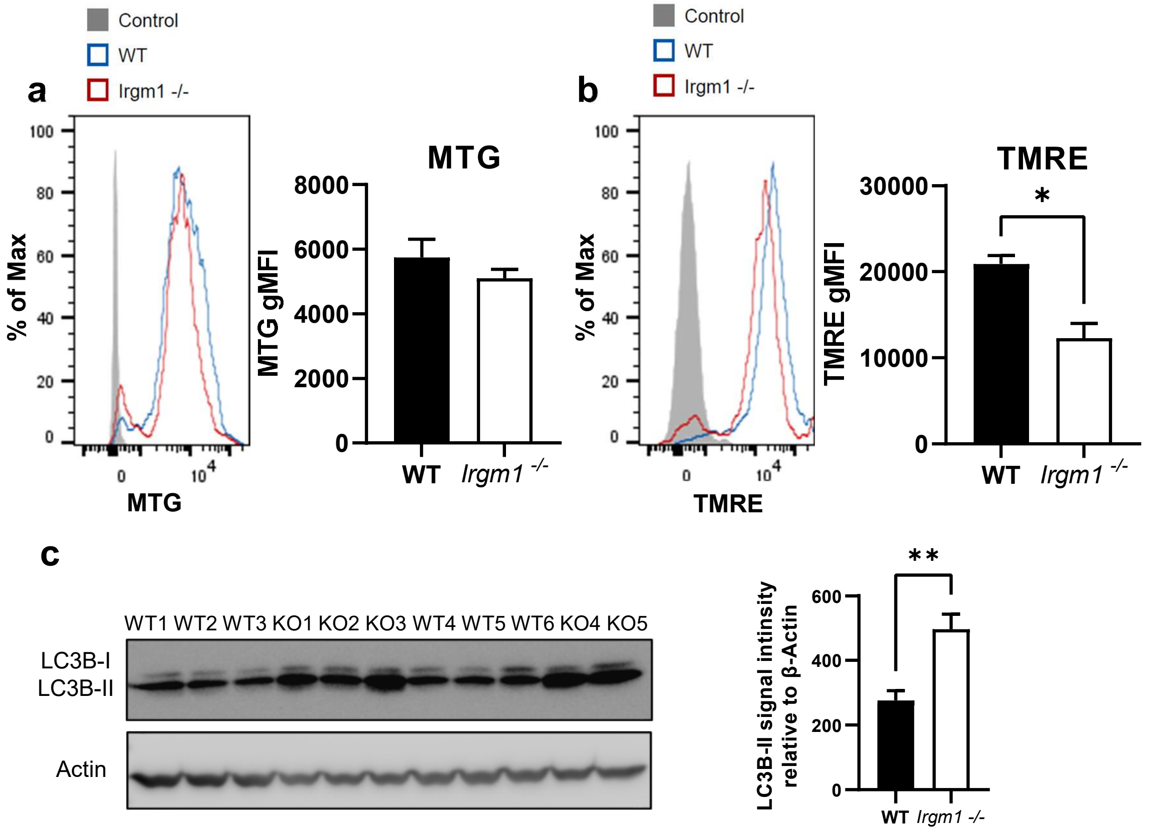

Supplementary Figure 4. Irgm1 deficiency is associated with reduced mitochondrial potential and increase in the autophagosome protein LC3B levels in CD8$^+$ T cells. CD8$^+$ T cells were isolated from WT and *Irgm1$^{-/-}$* mice and activated for 48 hours with plate bound anti-CD3 and anti-CD28 antibodies. The activated CD8$^+$ T cells were stained with Mitotracker green (MTG) *(a)* and TMRE *(b),* then quantified by flow cytometry (data representative of two independent experiments n=3). From the same experiment, a portion of the cells were lysed and immunoblotted for LC3B protein expression relative to actin *(c).* (Samples pooled from two independent experiments n=5-6). Error bars represent ±SEM. Normality was tested using the Shapiro-Wilk test and T-test with Welch's correction was used to compare groups. *$p<0.05$, **$p<0.01$.

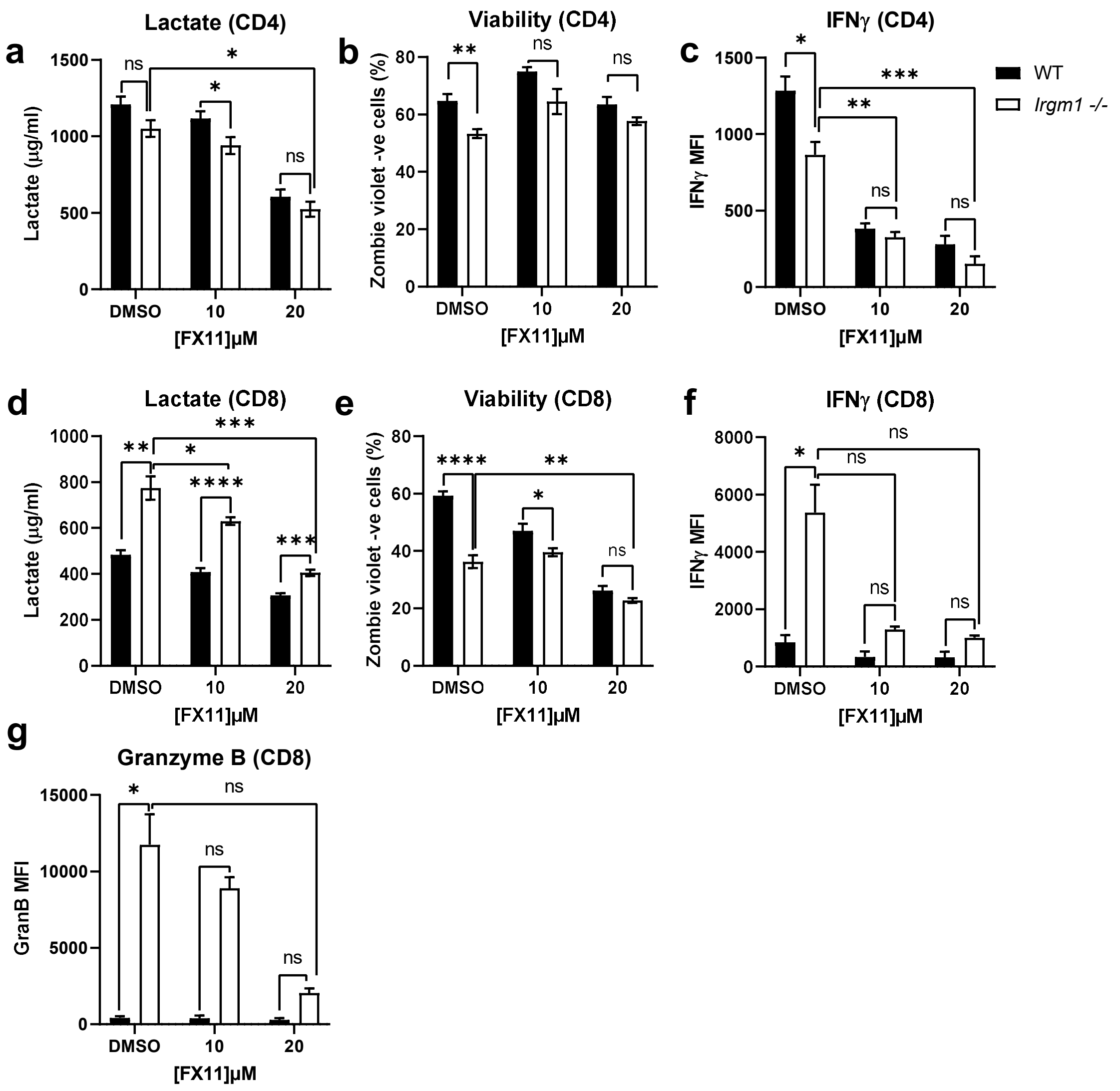

Supplementary Figure 5. Inhibition of lactate dehydrogenase in Irgm1 deficient T cells restores T cell viability and function. Isolated $CD4^+$ and $CD8^+$ T cells from WT and *Irgm1*$^{-/-}$ mice were activated in the presence or absence of FX11, after which media was collected and assayed for lactate *(a, d)* and cells were stained with Zombie violet dye to assess viability *(b, e)*, then fixed, permeabilized and stained for IFNγ *(c, f)* or Granzyme B *(g)*. Data pooled from two independent experiments (*n=4-6*); error bars represent ±SEM. Dunn's multiple comparisons test or multiple T-test with Welch's correction was used to compare groups pairs depending on the normality of the distribution as judged by the Shapiro-Wilk test.*p<0.05, **p<0.01, ***p<0.001, ****p<0.0001, ns, not significant.

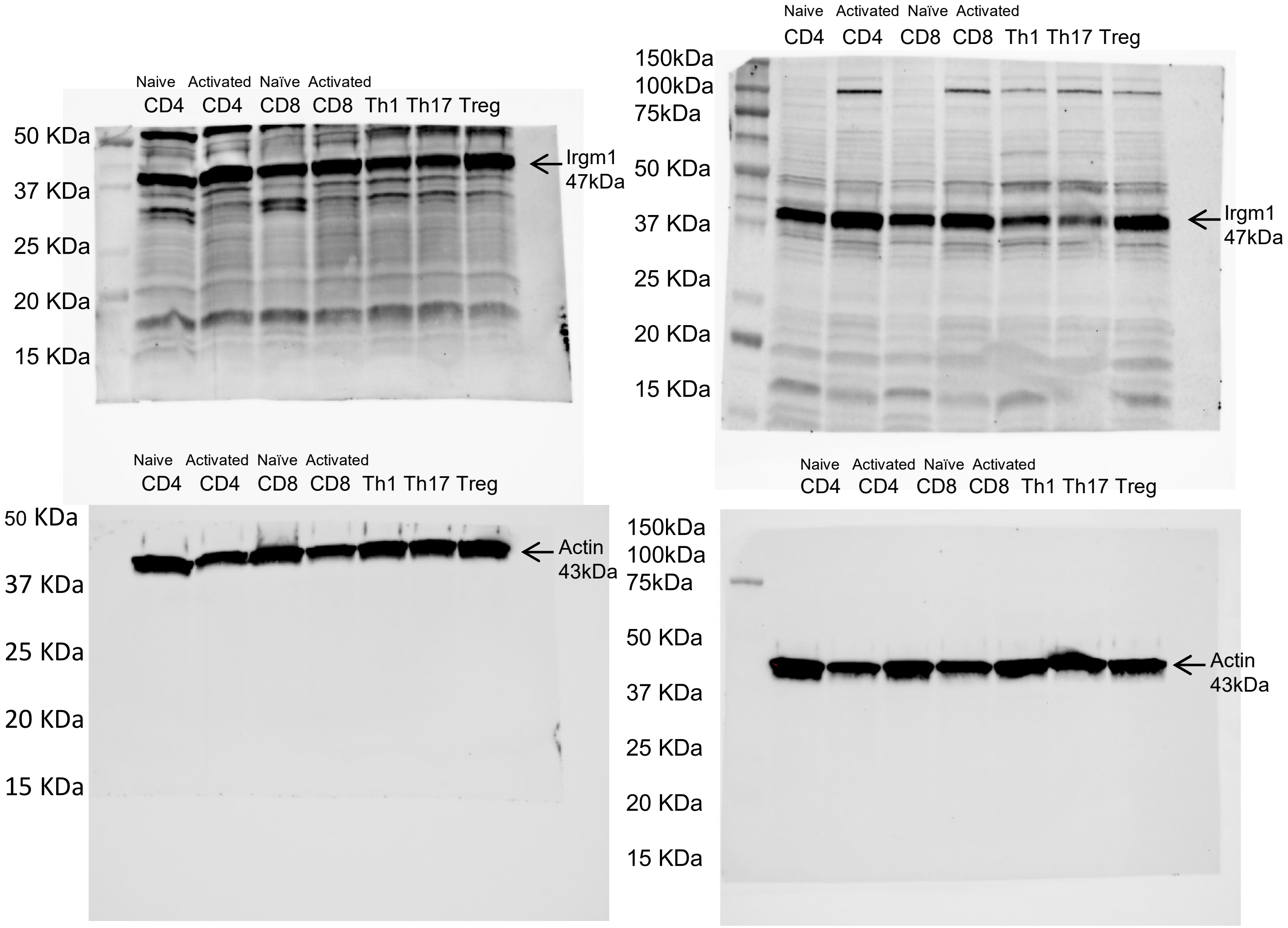

Original blots used to prepare Figure 1B

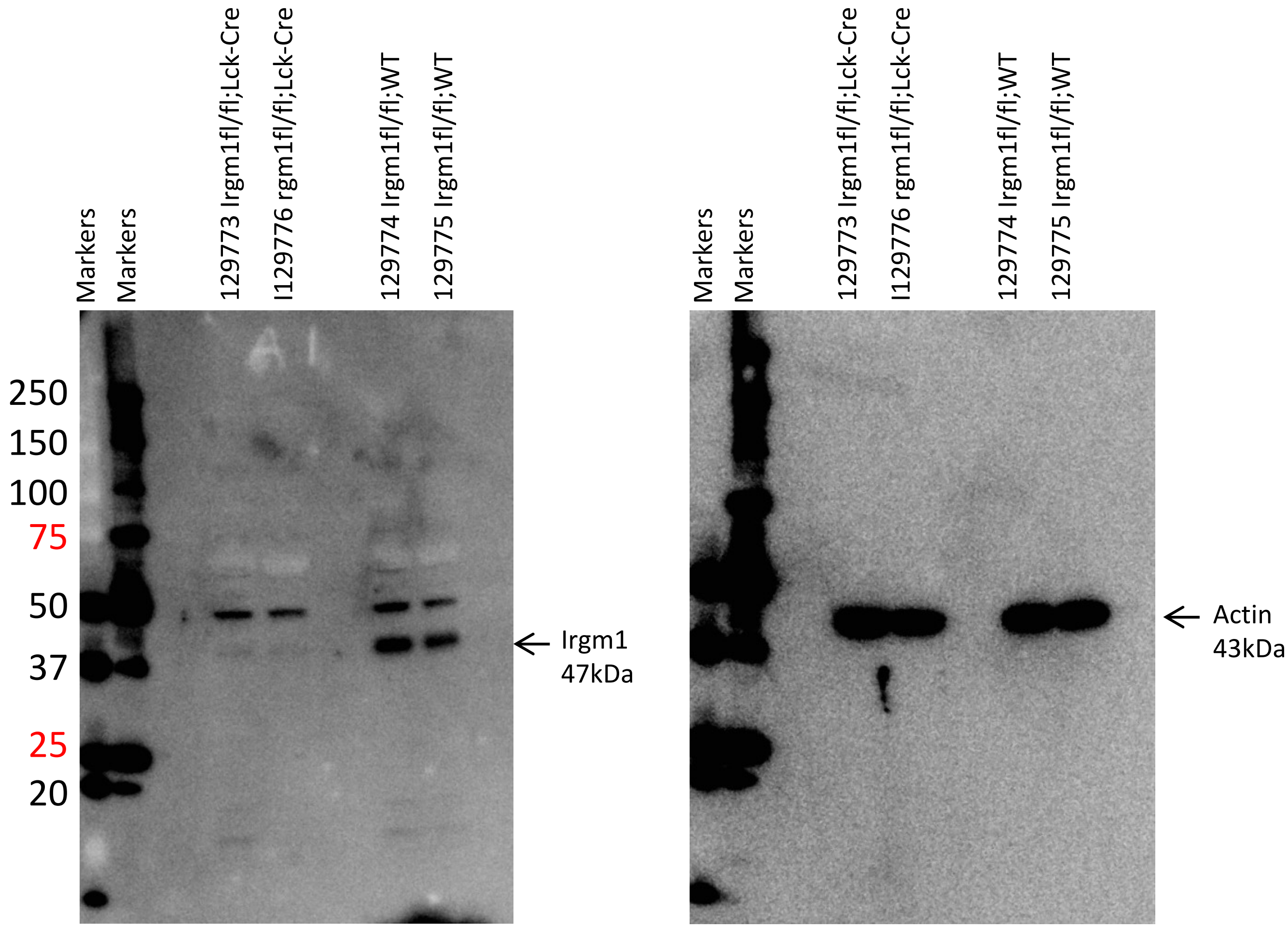

Original blots used to prepare Figure S3

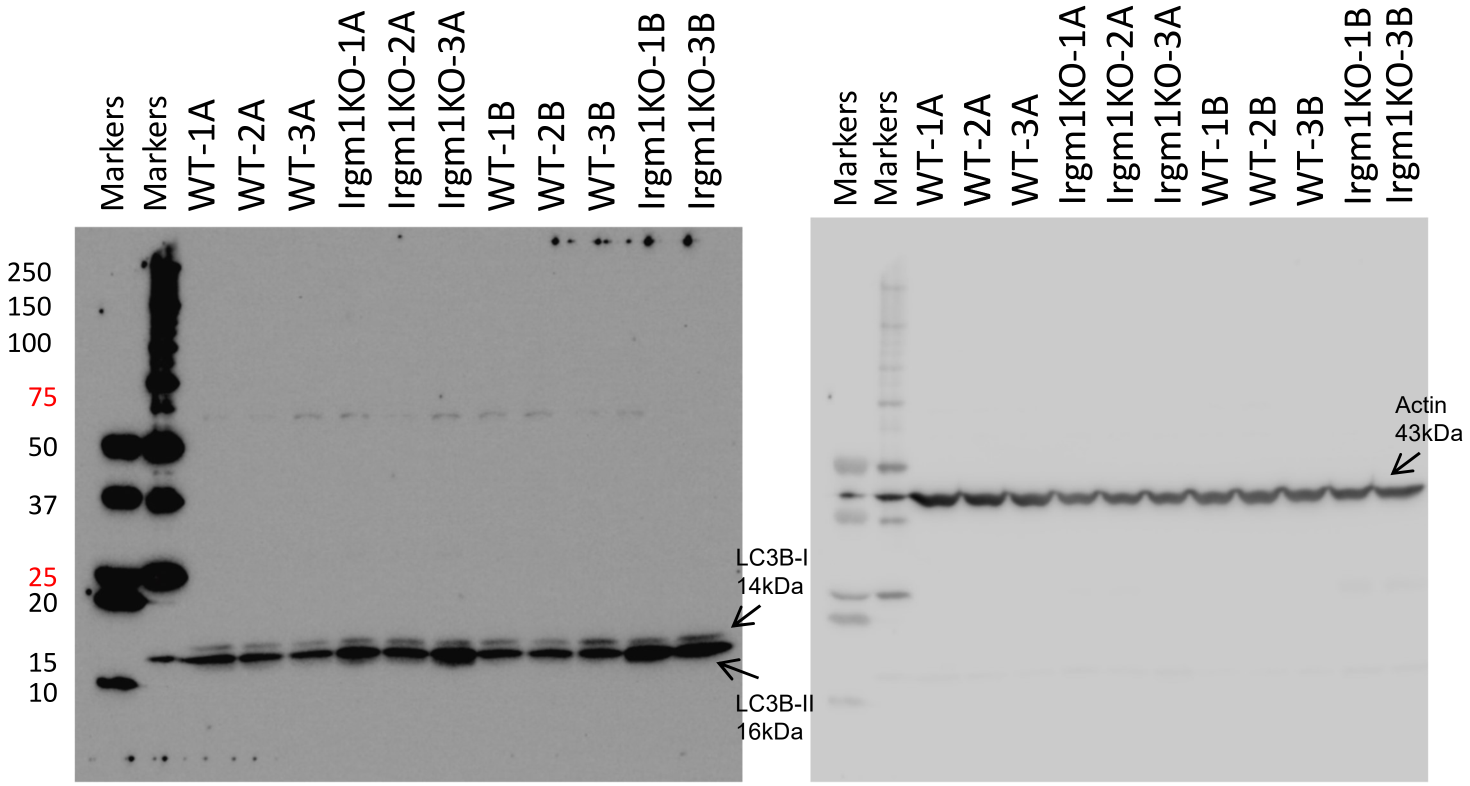

Original blots used to prepare Figure S4
